# Nano drugs delivery system: A novel promise for the treatment of atrial fibrillation

**DOI:** 10.3389/fcvm.2022.906350

**Published:** 2022-10-26

**Authors:** Zhengjie Wang, Qi Tong, Tao Li, Yongjun Qian

**Affiliations:** Department of Cardiovascular Surgery, National Clinical Research Center for Geriatrics, West China Hospital, Sichuan University, Chengdu, China

**Keywords:** nano drug delivery system, nano particles, arrhythmia, atrial fibrillation, treatment

## Abstract

Atrial fibrillation (AF) is one of the most common sustained tachyarrhythmias worldwide, and its prevalence is positively correlated with aging. AF not only significantly reduces the quality of life of patients but also causes a series of complications, such as thromboembolism, stroke, and heart failure, increases the average number of hospitalizations of patients, and places a huge economic burden on patients and society. Traditional drug therapy and ablation have unsatisfactory success rates, high recurrence rates, and the risk of serious complications. Surgical treatment is highly traumatic. The nano drug delivery system has unique physical and chemical properties, and in the application of AF treatment, whether it is used to assist in enhancing the ablation effect or for targeted therapy, it provides a safer, more effective and more economical treatment strategy.

## Introduction

Since the birth of nanotechnology, it's application in the medical field has brought appreciable enhancements in the accuracy of diagnosis and treatment of various diseases (1). Currently, more than 50 nanoparticle-based therapies are used for various indications, including infections, tumors, neurological diseases, ocular diseases, and cardiovascular diseases (2). In recent years, the application of nano drug delivery systems in the treatment of AF has also attracted much attention.

AF is a common persistent tachyarrhythmia. The global prevalence of AF currently ranges from 2% to 4%, and if screening of long-lived individuals and undiagnosed AF in the general population is expanded, the prevalence is expected to increase by a factor of 2.3 (3). There were approximately 37.6 million cases of AF worldwide in 2017 (4). As the incidence of AF increases with age, the absolute burden of AF is estimated to increase by more than 60% by 2050 as the global population ages (5). In principle, AF itself is not fatal, but this does not mean it is a benign disease; instead, AF has been reported to be associated with an increased risk of death in men (OR, 1.5 [95% CI, 1.2–1.8]) and women (OR, 1.9 [95% CI, 1.5–2.2]), (6) as it is a major factor in increasing the risk of fatal complications such as stroke, coronary heart disease, heart failure and thromboembolism, especially in advanced age (7, 8). The medical expenditures associated with the treatment of AF and its complications are estimated to exceed 28 billion dollars annually, placing a huge economic burden on society (9).

However, the current treatment strategies for AF are still intravenous drugs (e.g., diltiazem, metoprolol, and venakaran) and long-term oral drugs (e.g., flecainide, propafenone, and amiodarone) (10–13). In addition to poor efficacy and a high recurrence rate, there are often unavoidable toxic side effects. Furthermore, the long-term outcomes of surgery and catheter ablation are unsatisfactory. Therefore, it has become a major research focus in the field of AF treatment to find a therapeutic measure with good curative effects, few side effects and less recurrence.

This review aims to summarize some technologies based on nano drug delivery systems for the treatment of AF. From three main aspects, the main mechanism of AF and its treatment status, the introduction of nano drug delivery systems, and the application examples of nano drug delivery systems in the treatment of AF.

## Mainstream mechanisms in occurrence and maintenance of AF

**Figure 1:**
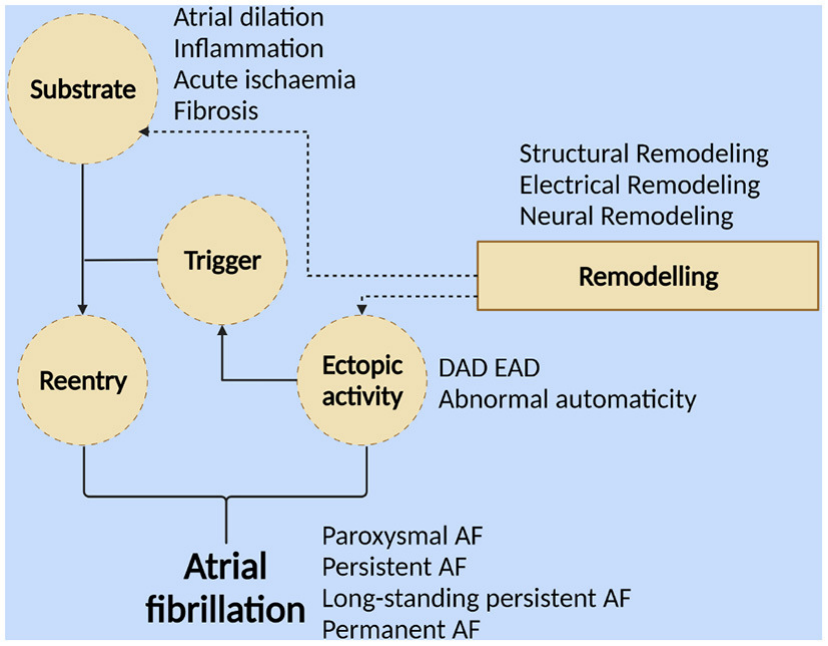
Different forms of atrial fibrillation result from ectopic activity and/or reentry. The formation of reentrants requires triggering activity and a matrix that is susceptible to reentrant formation. “Triggering” is a series of ectopic activities with abnormal autonomy (including early postdepolarization and delayed postdepolarization). Atrial dilation, inflammation, myocardial ischemia, fibrosis, etc., can form substrates that facilitate the formation of reentry. “Substrate” refers to persistent changes in structure, electrophysiological parameters, and innervation. Atrial remodeling, including electrical, structural, and neural remodeling, promotes the formation of this AF-prone substrate and the development of ectopic activity (Created with BioRender.com).

The mechanism of AF occurrence is complex and controversial, and the dominant mechanism varies among different individuals. The current core mechanisms (Figure 1) indicate that AF occurs when a series of rapid, disordered ectopic electrical activities replace the regular electrical activity of the sinus node. This ectopic electrical activity usually originates from the ectopic pacing focus of the pulmonary vein myocardial sleeve (PVs), where abnormal cardiomyocytes are structurally and electrophysiologically distinct from normal cardiomyocytes (14–16).

More than two-thirds of patients with recently diagnosed AF have a paroxysmal pattern, but between 5 and 10% per year progress to persistent AF. During 10 years of follow-up, more than half of patients with an initial diagnosis of paroxysmal AF converted to persistent AF or died (15).

The transformation of AF from paroxysmal to persistent or even permanent requires persistent ectopic activity or reentry. The occurrence of reentry requires the support of two conditions: the “triggering” activity (including early postdepolarization and delayed postdepolarization) and the “substrate” (persistent changes in structure, electrophysiological parameters, and innervation that contribute to the maintenance of AF) (14, 16–18). Atrial remodeling promotes the formation of this AF-prone substrate, which includes electrical remodeling characterized by long-term changes in atrial electrophysiological parameters, changes in current ion density of various ion channels, and changes in channel dynamics; structural remodeling characterized by atrial dilation, hypertrophy of atrial myocytes, changes in various organelles, and atrial fibrosis due to accumulation of collagen fibers; neural remodeling characterized by altered autonomic nervous system activity, increased sympathetic/parasympathetic fiber density, and altered numbers of muscarinic receptors (19, 20).

These remodeling processes promote each other and together form a substrate that is conducive to the maintenance of AF. Studies have shown that the mechanism behind the occurrence of atrial remodeling may be related to acute atrial ischemia, abnormal activation of the local renin-angiotensin-aldosterone system (RAAS) in myocardial tissue, inflammatory mediators (TNF, IL-2, CD36, HSP27, CRP), immune cell infiltration, cardiomyocyte apoptosis, etc. (21–28), and these mechanisms will provide theoretical support for the treatment of AF based on the nano drug delivery system described later.

## The main traditional treatment strategies for AF

### Classic antiarrhythmic drugs

The treatment of AF is still based on long-term oral or intravenous drugs, with the goal of restoring normal sinus rhythm, controlling ventricular rate, and preventing and treating related complications (11, 29). Several classic antiarrhythmic drugs such as sodium channel blockers or so-called polygon channel blockers (e.g., amiodarone) can be used to restore sinus rhythm, and β blockers and calcium channel blockers (e.g., verapamil) can slow down atrioventricular nodal conduction to achieve physiological ventricular rate.

### Catheter and surgical ablation

Catheter ablation, surgical ablation, and hybrid therapy have all been proposed as treatments for AF, but they vary in efficacy and accessibility. In recent years, transvenous catheter ablation of pulmonary vein isolation (PVI) has been the treatment of choice for symptomatic patients who require rhythm control and fail antiarrhythmic drug therapy (30–33).

Surgical ablation (SA) is often performed concurrently with other cardiac procedures by creating an electrically silent lesion group, passing electrical impulses through a narrow organized pathway or maze, interruption of major reentry, and restoration of sinus depolarization initiation (34–36). Since then, to further simplify the surgical procedure, shorten the operation time, improve the treatment efficiency, and reduce surgical complications, new surgical energy sources, such as radio frequency, cryo-balloon, ultrasound, and microwave, have been introduced (33, 37). Currently, hybrid therapy appears to be an optimal solution combining the respective advantages of catheter and surgical ablation, such as reliable conduction block effect, elimination of ablation gaps that can lead to long-term recurrence, and reducing the risk of potential surgical injury to hard-to-reach tissues (35).

### The limitation of traditional treatments

Classic antiarrhythmic drug treatment not only has poor curative effects and a high recurrence rate, but will also inevitably produce toxic side effects after long-term use (38, 39). Although there are various ablation strategies in the clinical practice of AF treatment, in general, neither endocardial catheter ablation nor surgical ablation has achieved satisfactory results in the success rate of AF elimination (3, 40–45), and there is a significant learning curve; experienced medical centers are far more effective than those with fewer deployments (46, 47). The CABANA trial showed that 36.4% of catheter ablation patients experienced recurrence of AF within 12 months after surgery, and 18.4% of catheter ablation patients developed symptomatic AF throughout follow-up (48). In terms of safety, although catheter ablation is recognized as a relatively safe treatment, its risks cannot be ignored. Based on prospective data from multiple registries, the overall incidence of complications in patients with AF undergoing catheter ablation is approximately 3–9%. The incidence of vascular injury and neurological complications is 0.2–5%, and the incidence of esophageal injury is less than 1% (49, 50). SA combined with other cardiac procedures significantly increased the requirement for pacemaker implantation (51), reported from 6.8 to 21.5% (3, 52). To find a treatment strategy with a high AF elimination rate, low recurrence rate, and high safety, various improvement methods have been explored in the field of heart disease treatment. Among them, the combination of nanotechnology and medical treatment has provided a new idea for the treatment of AF.

## Nano drug delivery system

Traditional systemic drug delivery methods are insufficient in terms of stability, solubility, and bioavailability, and have various adverse side effects. Amiodarone (AMD) is commonly used as a class III antiarrhythmic drug for the conversion of AF (53–55), but due to its accumulation in untreated tissues, especially the lung, spleen, and liver, it can cause serious adverse reactions, and can also lead to hypothyroidism or a risk of hyperactivity (56–58).These problems can be well-addressed by using nanotechnology-based methods.

Nano drug delivery systems refer to a family of nanomaterials with extremely small sizes, unique shapes and surface properties. Due to its special physicochemical properties and biological properties, it has received extensive attention in the medical field.

The basic structure of nanoparticles (NPs) used in drug delivery systems usually consists of a particle core, a biocompatible protective layer, and linking molecules. The drug to be delivered can be encapsulated in the particle core or bound to the particle core surface. The biocompatibility protective layer is used to improve the biocompatibility of the drug delivery system and plays the role of avoiding the inflammatory response, reducing immunogenicity, and preventing the drug from being destroyed prematurely. Linking molecules are a class of compounds with active ends, whose role is to link NPs with biologically active molecules.

The definition and classification of NPs has changed as the field continues to evolve. Generally, the size of NPs is between 1 and 100 nm. The US National Nanotechnology Program and the European Commission believe that the upper limit of nanoparticles cannot be limited to 100 nanometers. In the medical field, nanoparticles range in size from 5 to 250 nanometers. There are also nanosystems that may exceed a few microns in size, such as liposomes (59–61).

Particles commonly used to carry drugs mainly include the following: nanoparticles (NPs), liposomes (liposomes), solid lipid nanoparticles (SLNs), nanocells (NMs), microemulsions, nanosuspensions, etc. (62). Nano drug delivery systems have some unique advantages over traditional drugs due to their physical, chemical, and biological properties (extremely small size, large specific surface area, ability to achieve special biological functions through multiple chemical modifications).

### Drug protective effects

For oral drugs to enter the blood, they must first be dissolved in the gastrointestinal fluid. The protective layer on the surface of the carrier can prevent the drug from being destroyed by the physical and chemical environment of the body's digestive system and the action of various enzymes or recognized and destroyed by the immune system before reaching the point of action, at the same time, it can also prevent the unstable drug from being converted into active ingredients prematurely in the body, and avoid toxic reactions due to excessive active drug doses (63, 64). Taking advantage of this property can reconsider unstable and easily disrupted drugs for treatment, and can also enhance the bioavailability of those drugs that are greatly affected by presystemic metabolism in the gastrointestinal tract (65).

### Sustained release

In the sustained-release system, the protective layer of the drug carrier is designed in various release modes: shell that can be eroded/degraded, swelling and dissolving matrix, organic membrane permeation or inorganic porous hollow particles, etc. (57). It is released at a slow rather than constant rate over an extended period of time, prolonging the duration of action of the drug in the body (66). Reduce the frequency of medication, improve patient compliance, ensure that the drug stays at the effective concentration stably, avoid toxic and side effects caused by exceeding the therapeutic concentration, and improve the effectiveness and safety of the drug (63, 64).

### Controlled release

In a broad sense, controlled release refers to controlling the release rate, release location and release time of the drug. In a narrow sense, controlled release refers to a controlled release formulation that releases a drug at a zero-order or near-zero-order rate within a predetermined time (66). The triggering conditions for drug release can be artificially designed so that the drug is released in a desired form under the desired conditions. According to whether the triggering condition is inside or outside the human body, it can be divided into internal stimulation and external stimulation. Internal stimuli such as body temperature, pH value, enzymes, etc.; external stimuli such as externally applied magnetic fields, electric fields, ultrasonic waves, heating, light, etc. (67–69).

### Targeted drug delivery

Targeting can be divided into active targeting and passive targeting (70). By changing the distribution of drugs in the body, reducing the concentration of non-acting targets and increasing the concentration of active targets, the efficacy is improved while reducing toxicity and side effects. After the nano drugs delivery system enters the human body, most of it is captured by the reticuloendothelial system due to its extremely small size. Passive targeting methods, such as adjusting the shape, size, surface charge, and increasing hydrophilicity of nanoparticles, can allow them to enter long circulation, avoid aggregation in the reticuloendothelial system and non-specifically increase nanoparticles in diseased organ areas. In addition, nanoparticles can also achieve active targeting in programmed forms, such as using receptor-ligand binding, antigen-antibody binding, chemical affinity differences, and other mechanisms to achieve targeted delivery specifically to the intended tissue (61, 71–74). Nanocapsules have been widely used for their targeted delivery (75).

## Practical application of AF treatment method based on nano drug delivery system

Many studies have reported the application of nano-drug delivery systems in the treatment of AF (Table 1). This chapter will combine these practical applications to illustrate how nano-drug delivery systems can play a role in the treatment of AF.

**Table 1:** Studies of nano drug delivery system for the treatment of atrial fibrillation.

**Types of nanoparticles**	**Drugs used in the treatment of AF**	**Mechanism of therapeutic action**	**Characteristics of the delivery system**	**References**
Chitosan nanoparticles	Botulinum toxin	Reduces vagus-induced shortening of the ERP	Increases solubility and bioavailability; Drug protective effects; Sustained release	(76)
Poly-(lactide-co -glycolide)(PLGA) polymeric nanoparticles	*CaCl*_2_; L-glutamate	Suppresses GPs function; Increases neuron apoptosis in GP	Controlled release	(77)
Polylactic-co-glycolic acid nanoparticles	Budesonide	Inhibits the inflammatory response	Increases solubility and bioavailability	(78)
Niosomal carriers	Carvedilol	Inhibits *I*_*N*_*a* and *I*_*C*_*a* in a concentration-dependent manner	Increases solubility and bioavailability	(65)
Poly(lactic-co-glycolic acid)(PLGA)-magnetite nanoparticles	*CaCl*_2_	Suppresses GPs function; Increases neuron apoptosis in GP	Targeted drug delivery; Controlled release	(79)
Poly-N-isopropylacrylamide-co-acrylamide coated magnetite nanoparticles	N-isopropyl acrylamide monomer	Inhibition of glycolytic enzymes leading to toxic effects on both neurons and axons	Targeted drug delivery; Controlled release	(80)
Poly(lactic-co-glycolic acid) (PLGA) polymeric nanoparticles	Amiodarone	Non-competitive adrenergic blocking effect of the inactivated *Na*^+^ channels; Reduces the *Ca*^2+^ current, outward and inward rectifier *K*^+^ current	Controlled release; Sustained release	(63, 64)
N-isopropyl acrylamide coated magnetite nanoparticles	N-isopropyl acrylamide monomer	Reduce the activity of ganglion cells	Targeted drug delivery; Controlled release	(81)

### Improving drug stability, solubility, and bioavailability using a nano drugs delivery system

Spontaneous *Ca*^2+^ waves occur when calcium overload occurs in the sarcoplasmic reticulum and some pathological changes are combined. Such spontaneous *Ca*^2+^ waves induce cell membrane depolarization through the mechanism of *Na*^+^/*Ca*^2+^ exchange, causing arrhythmias and systolic dysfunction (82). Carvedilol (CRV) inhibits the production of spontaneous *Ca*^2+^ waves and thus inhibits the occurrence of arrhythmias and has been approved by the FDA for the treatment of arrhythmias, including AF (83). Due to the poor water solubility of CRV and the great influence of the presystemic metabolism of the gastrointestinal tract, only approximately 25% can eventually be absorbed. Previous studies have shown that positively charged nanoparticles are more likely to be ingested by negatively charged cell membranes, and bile salts can enhance the osmotic effect of niosomes on biological barriers. The charged modified bile salt niosomal carrier constructed by Gelareh et al. greatly improves the bioavailability of CRV, reduces drug toxicity, and improves efficacy and patient compliance (65).

Ganglion plexus (GPs) act as integrative centers for afferent information and efferent outflows of perceptual stimuli and are capable of modulating cardiac electrophysiological function through cardio-cardiac reflexes. The need for targeted ablation of the atrial GP emerged as GP stimulation resulted in increased triggering activity in the PV as well as increased dispersion of action potentials, which are considered factors in the maintenance of AF. Ablation of the GP results in changes in cardiac autonomic control, which multiple studies have linked to a reduced risk of AF recurrence (74).

Botulinum toxin (BoTN) is a neurotoxin that acts on neural synapses, blocking the release of acetylcholine-containing synaptic vesicles from the presynaptic membrane, reducing vagus-induced shortening of the atrial effective refractory period and preventing autonomic remodeling (84, 85). The use of BoNT to block GPs is temporary, and there is no permanent structural damage; such an effect is favorable.

Both animal experiments and human studies have shown that injection of botulinum toxin into the atrial fat pad can temporarily reduce the susceptibility of atrial tissue to AF induction and the effect of the vagus nerve on the atrial effective refractory period, potentially inhibiting the progression of autonomic remodeling. This effect can break the so-called “AF-induced AF” vicious cycle, preventing AF from progressing to persistent or even permanent AF (86, 87). Furthermore, therapeutic doses of botulinum toxin are safe with relatively few side effects (88). In a study of human epicardial fat pad injections of BoTN for the prevention of AF, no serious adverse effects were observed during 1 or even 3 years of follow-up (62).

There is a certain time delay in the effect of BoNT-blocking nerve effects, and the blocking effect is temporary (89); therefore, a method to accelerate the effect of BoNT and increase its duration is highly desired.

Animal studies have demonstrated that chitosan nanoparticles prolong the time of BoTN nerve block (71). Chitosan is a of the good candidate for drug delivery systems. As a non-toxic cationic polymer that can be degraded in the human body, it not only has high biocompatibility but also exhibits a high degree of resistance to cell membranes. Affinity. Chitosan has free amino groups and can be combined with various biologically active components, such as antigens, antibodies, and enzymes, which enables it to achieve various biological functions after processing. In the course of treatment, whether it is intravenous injection or *in situ* release, the drug is inseparable from the form of solution, while traditional linear chitosan is insoluble at physiological pH, but chitosan nanoparticles can be suspended in aqueous solution, and the resulting suspension can be assimilated into a solution (76), using chitosan as a component of the nano drugs delivery system can effectively protect the BoTN loaded into it while improving solubility, enhancing effect, prolonging effect time and reducing toxic side effects.

### Targeted therapy for AF using a nano drug delivery system

Studies have found that hyperactivity of the cardiac autonomic nervous system can induce AF, AF not only causes electrical remodeling, but also further enhances the activity of the cardiac autonomic nervous system, forming a vicious cycle of “AF promoting AF” (90). Therefore, interventions in the autonomic nervous system activity of the heart become a potential target for atrial fibrillation therapy.

Studies have shown that *CaCl*_2_ inhibits the autonomic nervous activity of the heart because under the action of various mechanisms, the concentration of calcium ions in nerve cells remains basically stable, which is of great significance for maintaining the integrity of the cell structure. If the intracellular calcium ions accumulate excessively, neurotoxicity will occur and induce neuronal apoptosis (91). Similarly, studies have also shown that N-isopropylacrylamide monomers can inhibit the neurons of the cardiac autonomic nervous system by inhibiting glycolytic enzymes such as enolase (80, 81).

Based on the above mechanism, an atrial fibrillation treatment strategy was developed (79–81), which encapsulated drugs (*CaCl*_2_,N-isopropylacrylamide monomer) in magnetic nanoparticles, and a piece of permanent magnet was placed on the surface of the epicardium. After the drug was released to the coronary artery through catheter intervention, the external magnetic field generated by the permanent magnet pulled the magnetic nanoparticles loaded with drugs to the prepositioned GPs (77) to achieve the function of targeted drug delivery.

### Controlled and sustained release of drugs

AMD has poor water solubility, and low bioavailability. The active metabolite of AMD (deethylamiodarone) formed after absorption enters the transintestinal hepatic circulation and can extend the elimination half-life to approximately 60 days. As a result, active metabolites tend to accumulate *in vivo*, which can cause damage to non-therapeutic target organs such as the lungs, liver, and thyroid, causing serious adverse effects such as chronic interstitial pneumonia or pulmonary fibrosis (53, 56, 57). Amira et al. encapsulated AMD into PLGA nanoparticles prepared from polylactic acid-hydroxyacetic acid, which have good bioavailability. These nanocarriers can allow the AMD encapsulated in the carriers to be slowly released *in vivo*, avoiding toxicity to non-therapeutic organs (63, 64).

Polymer hydrogels are a good medium commonly used for controlled drug release. Nanoparticle outer coatings based on polymer hydrogels can provide stable protection and can degrade under certain triggering conditions (specific pH and temperature).

Poly-N-isopropylacrylamide-co-acrylamide is a temperature-sensitive polymer gel that hydrates at ambient temperatures below body temperature and becomes hydrophobic at body temperature (79), according to which the nanocontrolled release system is designed, disintegrates the outer coating after entering the human body environment, and releases the drug carried inside. In addition, this critical temperature for disintegration can be altered by adding residues. If the critical temperature is designed to be high so that it does not naturally disintegrate, the disintegration of the outer layer can only be triggered when the local high temperature can be reached instantaneously, such as radiofrequency ablation. Encapsulating the aforementioned drugs acting on GP inside the drug carrier can provide a better ablation effect for radiofrequency ablation and reduce the recurrence rate of AF.

During radiofrequency ablation, due to the local high temperature, the myocardial tissue at the ablation site will be inflamed, and the perioperative inflammatory response will increase the risk of early postoperative recurrence of atrial fibrillation. The budesonide is encapsulated with polylactic-co-glycolic acid (PLGA) nanoparticles. In the process of radiofrequency ablation, the nano drugs delivery system is triggered at high temperature to release the budesonide carried inside and inhibit the inflammatory response at the ablation site. In addition, some untriggered nanoparticles can provide a sustained anti-inflammatory effect on the ablation site through the sustained release effect, effectively enhance the effect of radiofrequency ablation, and reduce the risk of postoperative atrial fibrillation recurrence (78).

Similarly, there is a nano drugs delivery system designed as a chain, with a liposomal drug carrier and a tail composed of three magnetic nanoparticles (67). Magnetic particle tails can convert electromagnetic energy applied by an external magnetic field (radio frequency) into mechanical energy and transfer it to the liposome membrane through a chain-like structure. Subsequently, the membrane structure of the liposome is broken, and the drug inside is released. This drug-controlled release strategy can also be used for adjunctive drug ablation in radiofrequency ablation.

### Hyperthermia-based treatment of AF

Magnetic nanoparticles have a hysteresis effect. When the ferrous core of the magnetic nanoparticles is exposed to the alternating magnetic field of the positive and negative electrodes, magnetic hysteresis loss will occur repeatedly, and a part of the electromagnetic energy will be irreversibly converted into heat energy (92). Thermal energy deposition occurs due to continuous hysteresis loss. However, superparamagnetic nanoparticles have no hysteresis effect and generate thermal energy through Neel relaxation and Brownian motion in an alternating magnetic field. Using this thermal effect of magnetic nanoparticles to treat diseases is called magnetofluidic hyperthermia (MFH). The earliest reported clinical application of MFH was the ablation of sentinel lymph nodes in metastatic tumors.

Clinically, radiofrequency, ultrasound and other technologies are used to increase tissue temperature through thermal effects, thereby causing irreversible damage to the target, and are used to treat arrhythmias such as AF. MFH can also be used for ablation of AF. MFH guided by the magnetic particle imaging (MPI) technique for imaging using the properties of superparamagnetic nanoparticles is currently being investigated (93, 94). The combination of these two technologies can not only monitor the scope of ablation, but also display the temperature of the ablated tissue and the degree of tissue damage (95), which can estimate the effect of ablation, improve the ablation effect of AF and reduce the recurrence rate while ensuring safety.

In addition, if hyperthermic heating of cells is insufficient, polymer coatings with higher critical temperatures can also be designed to release endotoxins by heating the particles with an alternating magnetic field, thereby providing additional targeted killing for drug delivery (96, 97).

### Nano drugs delivery systems control substance distribution in the body

In addition to carrying synthetic exogenous substances, nano drugs delivery systems can also be used to change the distribution of endogenous substances in the human body.

Exosomes are a type of extracellular vesicles, usually 40–200 nm in size, that can carry various signaling biomolecules [including proteins, nucleic acids, and especially non-coding RNAs (ncRNAs)] inside and have the ability to mediate cell-to-cell interactions and exchange information between cells over long distances (98, 99).

In recent years, studies have shown that exosomes are involved in some important links in the pathogenesis of AF, and some ncRNAs have also been proven to be important factors in regulating cardiac function and participating in the occurrence and development of the disease, which has the potential to be used in the treatment of cardiovascular diseases (100–102).

Studies have shown that myocardial fibrosis in AF is associated with macrophages infiltrating into cardiac tissue, and macrophages lead to myocardial fibrosis through interactions with cardiomyocytes or fibroblasts, which in turn provide the basis for the development and maintenance of AF. Inhibition of “canonical” activated macrophage (M1) polarization and enhancement of “alternate” activated macrophage (M2) polarization improves myocardial fibrosis and alleviates myocardial remodeling (103).

Several studies (104) found reduced levels of miR-23a in exosomes derived from Ang II-treated atrial myocytes. The mechanism is the inhibition of nuclear translocation of NFATc3 through the non-coding repressor of NFAT (NRON), which in turn inhibits the expression of miR-23a (105). After these miR-23a-deficient exosomes are taken up by macrophages, they promote M2 macrophage polarization and can inhibit atrial myocyte fibrosis and have potential for AF therapy (103, 104, 106). MiR-126, miR-425, and miR-744-enriched exosomes are captured by cardiac cells and can act on pathways related to TGF-β and type I collagen (107–110) to inhibit fibrosis. MiR-17- and miR-210-enriched exosomes from cardiac progenitor cells are taken up by cardiac cells and act on TGF-β-related pathways to enhance tolerance to fibrosis under oxidative stress (111). Cardiomyocyte-derived exosomes enriched in miR-378, miR-29a, miR-29b, and miR-455 exert antifibrotic effects by reducing collagen and MMP9 by inhibiting MAPK and Smad pathways (112).

Cardiomyocyte apoptosis can occur before atrial remodeling, and AF can aggravate apoptosis. It can be seen that cardiomyocyte apoptosis and AF are mutually aggravating processes. Exosomes enriched in miR-210, miR-133a, and miR-19a reduce apoptosis by enhancing cardiomyocyte resistance to oxidative stress (113), and cardiomyocyte-derived miR-146a-enriched exosomes reduce scarring after myocardial infarction in rats (114), inhibit cardiomyocyte apoptosis and prevent cardiac remodeling. Exosomal miR-320d from adipose tissue-derived mesenchymal stem cells (MSCs) was shown to inhibit AF-induced cardiomyocyte apoptosis in a STAT3-dependent manner (115). Overexpression of miR-520d participates in the ability to regulate cell survival and apoptosis by targeting ADAM10 (116), ultimately leading to inhibition of apoptosis and induction of atrial myocyte survival, thereby ameliorating the vicious cycle in which cardiomyocyte apoptosis and AF mutually promote.

Acute atrial ischemia can aggravate the vulnerability of AF, and exosomal miR-19a from MSCs can reduce the expression of inflammatory cytokines. The immune response has also been shown to be involved in the pathogenesis of AF, and exosomal miR-19a from MSCs can reduce the expression of inflammatory cytokines, inhibit the inflammatory response and prevent the progression of AF (117).

Mechanisms based on these specific ncRNA-rich exosomes that inhibit fibrosis, resist apoptosis, suppress immune inflammatory responses, and promote angiogenesis are promising in the prevention and treatment of AF. However, insufficient retention of exosomes in the myocardium and the mechanism of endogenous manipulation of exosome biodistribution are still unclear, which are the main challenges in the clinical application of exosomes.

Nano drugs delivery systems for exosome binding have been developed to enhance the enrichment and retention of exosomes in tissues of interest, which can be applied for targeted delivery of exosomes to myocardial tissue. Studies (118) found that exosomes (myocardial infarction exosomes) present in the circulating blood after myocardial infarction have a protective effect on ischemic myocardium, and this protective effect is mediated by HSP70 on the surface of the exosome membrane and depends on the biodistribution of endogenous exosomes. However, another study (119) used magnetic nanoparticles to capture myocardial infarction exosomes in peripheral blood and targeted delivery to ischemic myocardium under the guidance of an external magnetic field, inhibiting ischemic myocardium fibrosis. This means that the use of magnetic nanoparticle targeting technology provides the possibility for exosomes in the treatment of AF.

## Conclusions and future direction

The application of nano drugs delivery system provides a new idea for the treatment of AF. Nonetheless, there are still some important limitations and challenges in the use of nano drugs delivery systems in the clinical treatment of AF.

From the point of view of AF treatment, because important structures in the heart, such as blood vessels, nerves, valves, and cardiac conduction systems, are adjacent to each other, off-target effects during the treatment process will result in a serious adverse prognosis. Future research needs to take into account the potential off-target risk of the treatment process and screen for methods with high accuracy and efficiency. In the application of MFH ablation of AF, there are symptoms such as pain and muscle twitching caused by external magnetic field stimulation of peripheral nerves (120), which puts forward further requirements for the safety of this technology.

Furthermore, due to the heterogeneity of nanodrug delivery systems in humans, some nanoparticles undergoing preclinical development have been retrospectively found to be cytotoxic or immunogenic (121, 122). With the application of magnetic nanoparticles, the iron core of magnetic nanoparticles was initially thought to be able to enter the normal iron cycle of the human body, but it is not clear whether excessive accumulation of iron occurs after long-term use. Studies have shown that excessive accumulation of iron can cause iron overload-induced apoptosis (123), which cannot be ignored. More research is needed to provide evidence for optimizing biocompatibility and antifouling, reducing exposure to disturbances in biological systems, and exploring safety for long-term use.

From the perspective of nano drugs delivery system production, how to maintain a certain degree of stability in the physical and chemical properties, encapsulation rate, release rate, etc., of the nano drug drugs delivery system in the process of large-scale repeated production, that is, to ensure that the entire production process has a high degree of repeatability and reliability, how to reduce the loss of the produced nano drugs delivery system during storage and transportation, and how to reduce the economic cost and time cost of production. These questions all relate to the possibility of applying this technology to the clinic.

## Author contributions

ZW, QT, TL, and YQ prepared the manuscript. ZW, QT, and YQ wrote the main parts of the article and produced graphics. TL, QT, and YQ reviewed and edited the manuscript. ZW and YQ drafted the final version of the manuscript. All authors read and approved the final manuscript.

## Funding

YQ acknowledges lab support provided by grants from the Scientific Research Project of National Clinical Research Center for Geriatrics, West China Hospital, Sichuan University (No. Z2018B19), and 135 project for disciplines of excellence–Clinical Research Incubation Project, West China Hospital, Sichuan University (No. 2019HXFH029), and Technology innovation research and development project of Chengdu Science and Technology Bureau(No. 2019YF05-00183-SN), and the Major Science and Technology Project of Sichuan Province, China (No. 2021YFS0121), and the Science and Technology Project of Sichuan Province Health Commission, China (No. 21PJ035).

## Conflict of interest

The authors declare that the research was conducted in the absence of any commercial or financial relationships that could be construed as a potential conflict of interest.

## Publisher's note

All claims expressed in this article are solely those of the authors and do not necessarily represent those of their affiliated organizations, or those of the publisher, the editors and the reviewers. Any product that may be evaluated in this article, or claim that may be made by its manufacturer, is not guaranteed or endorsed by the publisher.
